# Rapid and sensitive large-scale screening of low affinity extracellular receptor protein interactions by using reaction induced inhibition of *Gaussia* luciferase

**DOI:** 10.1038/s41598-020-67468-7

**Published:** 2020-06-29

**Authors:** Francis Galaway, Gavin J. Wright

**Affiliations:** Cell Surface Signalling Laboratory, Wellcome Sanger Institute, Cambridge, UK

**Keywords:** Membrane proteins, Cell adhesion

## Abstract

Extracellular protein interactions mediated by cell surface receptors are essential for intercellular communication in multicellular organisms. Assays to detect extracellular interactions must account for their often weak binding affinities and also the biochemical challenges in solubilising membrane-embedded receptors in an active form. Methods based on detecting direct binding of soluble recombinant receptor ectodomains have been successful, but genome-scale screening is limited by the usual requirement of producing sufficient amounts of each protein in two different forms, usually a “bait” and “prey”. Here, we show that oligomeric receptor ectodomains coupled to concatenated units of the light-generating *Gaussia* luciferase enzyme robustly detected low affinity interactions and reduced the amount of protein required by several orders of magnitude compared to other reporter enzymes. Importantly, we discovered that this flash-type luciferase exhibited a reaction-induced inhibition that permitted the use of a single protein preparation as both bait and prey thereby halving the number of expression plasmids and recombinant proteins required for screening. This approach was tested against a benchmarked set of quantified extracellular interactions and shown to detect extremely weak interactions (*K*_D_s ≥ μM). This method will facilitate large-scale receptor interaction screening and contribute to the goal of mapping networks of cellular communication.

## Introduction

Proteins embedded in the cellular membrane are critically placed to transmit signals from the external environment to the interior of the cell to ensure coordinated and appropriate cellular behaviours from the earliest stages of development to the maintenance of adult tissues^1^. Importantly, these cell surface proteins are targets for therapeutic antibodies that are increasingly used to treat indications such as autoimmunity and cancer^2^. Despite their medical relevance, however, the specialised biochemical nature of membrane proteins has meant that they are often underrepresented in large-scale protein interaction screens^3^ which typically employ techniques such as biochemical purifications and transcription-based protein complementation assays^4^. Biochemically, membrane proteins are difficult to solubilise in their native conformation due to their amphipathic character, and the interactions between cell surface receptor proteins can be extremely transient—having dissociation half-lives of just fractions of a second—making protocols that include stringent wash steps unsuitable to detect them^4,5^.

Several approaches that address these biochemical challenges have been developed (reviewed recently in^6^) and include mass spectrometry-based techniques for association by proximity labelling^7,8^, genetic manipulation of cell surface protein repertoires in the context of a cell membrane^9–11^, and expression cloning^12–14^. A very successful approach has been the development of ELISA-style assays that detect direct binding events within large libraries of soluble recombinant receptor ectodomains^15–17^, including the AVEXIS (AVidity-based EXtracellular Interaction Screening) method developed by our laboratory^18^. This general approach relies on the ability to express the extracellular binding domain as a secreted recombinant protein that retains the extracellular binding activity, and has been particularly useful for common receptor architectural classes such as type I, type II, and GPI-anchored membrane proteins. It is important that structurally critical posttranslational modifications such as glycosylation and disulfide bonds are retained, and so eukaryotic protein expression systems such as mammalian cells are most commonly used. Usually, the receptor ectodomains are produced in two forms: a “bait” protein that can be immobilised on a solid substrate such as a microtitre plate, and a soluble “prey”. To circumvent the low binding affinities, the prey proteins are purposefully oligomerised to create highly avid binding probes^4^.

While these methods are highly specific and sensitive, a major barrier to increasing their scale as well as their wider adoption by other laboratories has been the large resource requirements to create large libraries containing hundreds of different proteins suitable for interaction screening. For each candidate receptor, two separate protein expression plasmids are required to produce both the bait and prey protein preparations. Also, in comparison to other methods, mammalian protein expression systems are expensive and often low-yielding, and because the number of interactions to be tested increases exponentially with the number of proteins screened, the large amount of protein required for screening is often a limitation. These constraining factors have meant that most extracellular interaction screens of this type have been limited to just a few hundred proteins^15,16,18–20^; however, mammalian genomes are known to contain up to 2000 receptors testable using this approach^21–24^. In principle, the required resources would be significantly reduced by developing an assay format where the receptor ectodomain can be used as both a bait and as a prey, halving the number of expression plasmids, transfections, and protein handling steps. In addition, significantly reducing the amount of protein required for each binding test would remove another limitation to increasing the scale of the screens.

One approach to reduce the amount of protein required could be to use a highly sensitive enzyme as the binding reporter. Previously, enzymes including alkaline phosphatase and beta-lactamase have been used, but enzymes with higher catalytic rates, such as horseradish peroxidase (HRP), and those that generate light such as luciferases are potentially more sensitive for an in vitro assay. Light-generating luciferase enzymes are very sensitive and have been developed for expression and secretion by mammalian cells^25,26^. These include aquatic luciferases that can produce blue light in an extremely bright but transient “flash” and have been further developed to produce long-lasting “glow” characteristics^27^. Conceptually, those enzymes that can be irreversibly inactivated with an inhibitor provide the option of using the same enzyme in a binding assay; for example, an enzyme-conjugated protein could be immobilised, its enzyme activity inhibited, and the interaction to another protein tested using the same enzyme as a binding reporter. The development of such an assay could result in the need for just a single protein expression plasmid and protein preparation thereby significantly reducing the amount of resources required for large scale extracellular protein interaction screening involving thousands of proteins.

Here, we have explored the use of different enzyme reporters for low affinity extracellular protein interaction screening using an ELISA-style direct binding assay with the aim of reducing the amount of protein required, and investigate the possibility of using just a single recombinant protein construct for each protein tested. We report that highly avid constructs based on a flash luciferase—because the luciferase activity was inhibited by exposure to the substrate—enables the enzyme to be used as both bait and prey.

## Results

### Concatenated luciferases are highly expressed as part of an oligomeric binding probe

**Figure 1:**
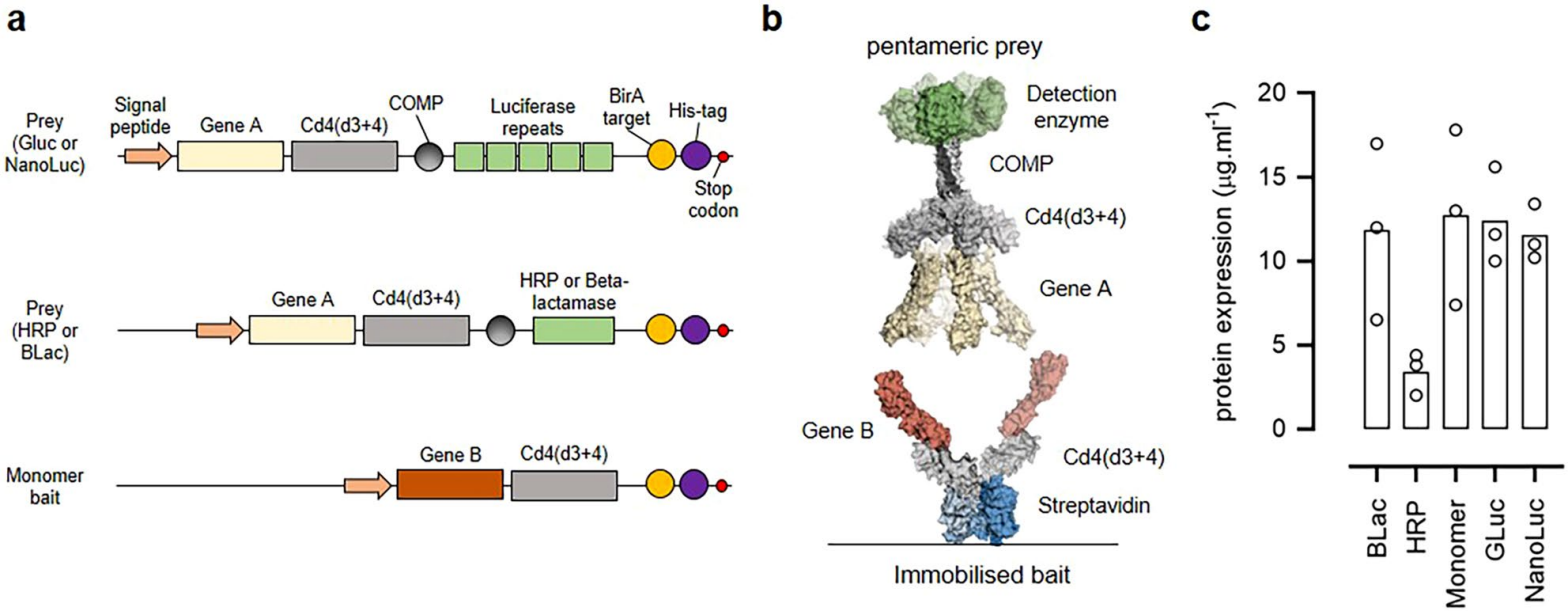
Design and expression of prey constructs with different reporter enzymes. (**a**) Schematic diagrams of the protein constructs used in this study. Prey proteins consisting of the extracellular region of a protein of interest (Gene A) are fused at their C-terminus to the proteins tags: rat Cd4(d3 + 4) and the rat cartilage oligomeric matrix protein (COMP) followed by either five repeating units of luciferase, HRP, or beta-lactamase enzymes. Baits are expressed as monomers and consist of ectodomains of a protein of interest (Gene B) fused to the same rat Cd4(d3 + 4) tag but additionally containing a peptide sequence that is recognised by the enzyme BirA for the covalent addition of a single biotin molecule. Both baits and preys contain a terminal 6-his tag for purification. (**b**) Schematic representation of the AVEXIS assay involving a soluble highly avid pentameric enzyme-tagged prey protein and biotinylated bait protein immobilised on a streptavidin-coated microtitre plate. (**c**) Pentameric preys containing the HRP enzyme were expressed at low levels. Rat Cd200 prey constructs containing the named enzyme reporters were expressed by transient transfection of HEK293 cells and the secreted protein yield quantified after nickel affinity purification. Data points are values for three independent transfections and bars represent the means. BLac = beta-lactamase prey; GLuc = *Gaussia* luciferase prey; NanoLuc = Nano luciferase prey.

In the original AVEXIS method, the receptor ectodomains were expressed as soluble recombinant proteins containing a C-terminal antigen tag consisting of the rat Cd4 protein, a protein sequence from the cartilage oligomeric matrix protein (COMP) that produced highly-avid pentamers, and the enzyme beta-lactamase^18,28^ (Fig. 1a, b). With the aim of reducing the amount of protein required in this assay, we first tried to replace the beta-lactamase enzyme with more sensitive reporter enzymes including HRP^29^, and five concatenated units of either a “glow”-type nano-luciferase^27^ and “flash”-type *Gaussia* luciferase^26,30^ (Fig. 1a). To enable protein quantitation and purification, both a 6-histidine tag and a peptide sequence that is a substrate for the protein-biotin ligase which permits enzymatic biotinylation were included at the C-terminus (Fig. 1a). To determine the level at which these new prey constructs were produced, protein expression plasmids were transfected into HEK293 cells, and the supernatant containing the secreted protein harvested, purified, and quantified. We observed that both the *Gaussia* and nano-luciferase prey constructs were expressed at the same high levels as the beta-lactamase prey (Fig. 1c); by contrast, we repeatedly failed to detect expression of the prey protein encoding HRP (Fig. 1c). One possibility for this lack of expression is that the supply of the necessary heme co-factor was limiting^31^; however, supplementing the cell culture medium with hemin failed to improve expression, and so we did not pursue HRP as a reporter enzyme any further.

### Low affinity extracellular interactions can be sensitively detected using luciferase reporter enzymes

**Figure 2:**
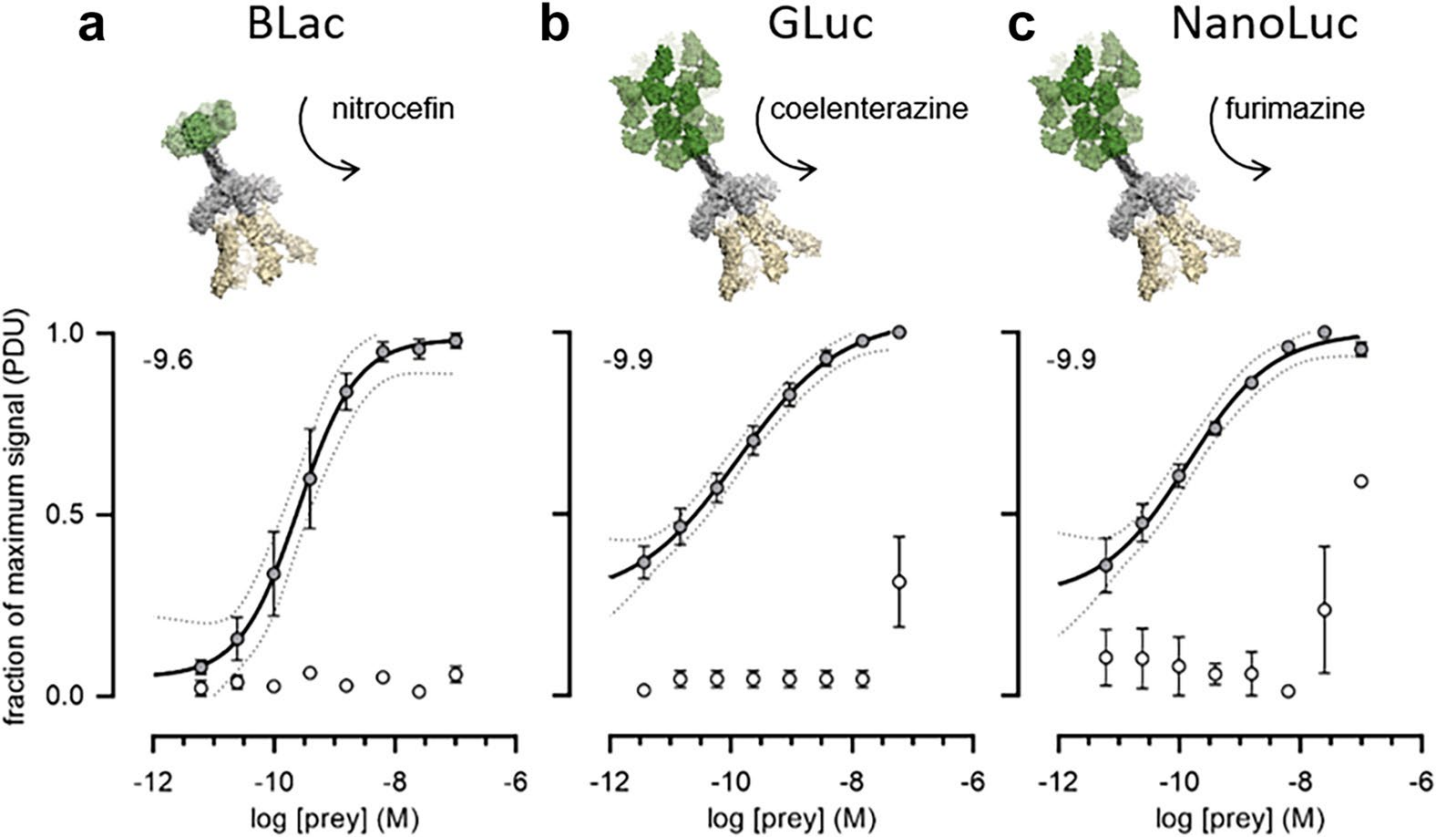
Luciferase reporters provide highly sensitive detection of the Cd200-Cd200R interaction. Highly avid rat Cd200 prey proteins containing the reporter enzymes (**a**) beta-lactamase (BLac), (**b**) *Gaussia* luciferase (GLuc) or (**c**) nano-luciferase (NanoLuc) were probed for interactions with a biotinylated rat Cd200R bait protein immobilised in streptavidin-coated microtitre plates. Prey capture was quantified using absorbance at 485 nm for hydrolysis products of the beta-lactamase colourimetric substrate nitrocefin, and luminescence using the substrates coelenterazine (*Gaussia* luciferase) and furimazine (nano-luciferase). Cd200 was used as a negative control bait (empty circles). The normalised signal for each data point was calculated as described in the Methods such that background signal would be 0 and the maximum signal 1. The log IC50 for each interpolated curve is displayed on each graph. Representative experiments shown with *n* = 3 for each concentration of prey with s.e.m. for each data point and the 95% CI for the interpolated curves indicated as dotted lines.

To quantify the ability of each prey construct to detect low affinity interactions, we prepared a dilution series of each prey and probed them against a fixed quantity of monomeric enzymatically mono-biotinylated bait immobilised in a streptavidin-coated plate. We used a typical low affinity extracellular protein interaction (rat Cd200-Cd200R) which has an equilibrium dissociation constant of 2.5 μM and a dissociation half-life of 0.9 s^32^. We observed that both the *Gaussia* and nano-luciferases provided a more sensitive detection of the interaction compared to beta-lactamase whilst maintaining a low background signal (Fig. 2). In fact, even at the lowest concentration tested, there was still a robust signal that was clearly discriminated from the control, and especially using the *Gaussia* luciferase.

### Reaction-induced inhibition of Gaussia ‘flash’ luciferase permits single construct use as both bait and prey

**Figure 3:**
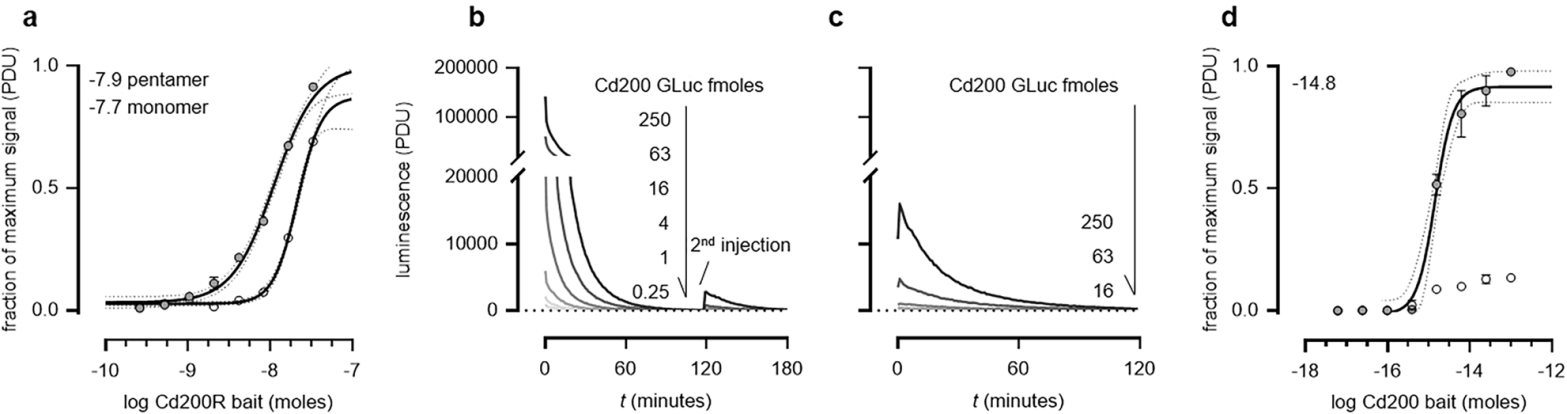
Reaction-induced inactivation of *Gaussia* luciferase enables the use of a single construct as both a bait and a prey. (**a**) Pentameric prey proteins can be used as a bait for sensitive interaction detection. Biotinylated Cd200R *Gaussia* luciferase-tagged pentamer (filled circles) or monomers (empty circles) were serially diluted and immobilised on a streptavidin-coated microtitre plate as bait proteins and probed for interactions with a beta-lactamase-tagged Cd200 pentamer. (**b**) Coelenterazine was added to a dilution series of purified Cd200 *Gaussia* luciferase pentamer which elicited a strong luminescence signal that was gradually lost after around an hour. A second administration of substrate did not generate the same bright luminescence signal. (**c**) *Gaussia* enzymatic activity is not restored after a nickel-affinity purification of the inactivated luciferase. Reaction inhibited *Gaussia* luciferase was purified using Ni–NTA agarose beads with several wash steps before addition of fresh coelenterazine (100 pmol). (**d**) Pentameric (non-biotinylated) Cd200 *Gaussia* luciferase prey was probed against biotinylated and inactivated pentameric Cd200R *Gaussia* luciferase bait (filled circles). The Cd200 prey was also probed against a reaction-inhibited pentameric *Gaussia* luciferase Cd200 protein used as a non-interacting control bait (empty circles). The inflection point for each interpolated curve is displayed as the log baits (moles) value on each graph. Representative experiments shown with *n* = 3 for each quantity of prey with SEM for each data point and the 95% CI for the interpolated curves.

With the ultimate aim of using the same ectodomain-containing protein expression construct as both a bait and a prey, we first asked whether a highly avid prey protein could substitute as a bait in our assay. We immobilised biotinylated Cd200R *Gaussia* luciferase pentamers on streptavidin-coated microtitre plates and probed them for interactions with a Cd200 beta-lactamase tagged prey. We observed that the interaction could be robustly detected and the sensitivity of the assay increased when compared to immobilised monomer baits (Fig. 3a).

To use the same protein as both a bait and a prey, it will be necessary to inhibit the enzymatic activity of the immobilised protein, and so we asked whether the “flash” activity of the *Gaussia* luciferase led to the irreversible inhibition of the enzyme. For the pentameric prey constructs, this would require inactivation of the luciferase enzyme reporter when arrayed as a bait such that the luminescent activity is reduced by > 90% for the remaining duration of the assay (~ 2 h) and is not dependent upon a labile or soluble inhibitor that would be removed during wash steps. We observed that once the coelenterazine substrate is added to the *Gaussia* luciferase enzyme immobilised on the microtitre plate, after an initial very bright burst of light, there is a gradual decline in luminescence that becomes undetectable after an hour (Fig. 3b). Subsequent additions of coelenterazine substrate did not result in further increases in luminescence demonstrating a reaction-induced inhibition of the enzyme (Fig. 3b). To determine if this inactivation was due to a labile reaction-induced inhibitor that would be removed during assay washing steps, the coelenterazine-treated luciferase-containing prey proteins were re-purified using their 6-his tags by Ni–NTA affinity chromatography. By adding coelenterazine substrate a second time to these purified enzymes, we observed that the inactivation of the *Gaussia* luciferase enzyme was not reversed (Fig. 3c). Together, these results demonstrate that the inhibition of the *Gaussia* luciferase enzyme cannot be easily reversed by wash steps and so will not interfere with the subsequent application of the prey reporter in an interaction assay.

The substrate-dependent inactivation of *Gaussia* luciferase therefore provided an opportunity to use the same protein expression plasmids to produce both the biotinylated bait and soluble prey. To determine if reaction-inhibited *Gaussia* luciferase Cd200R pentamer could be used as a bait, it was expressed as a biotinylated protein and a dilution series was immobilised in wells of a streptavidin-coated plate. Coelenterazine was added to inactivate the luciferase enzyme in the immobilised protein, and then each well was probed with the non-biotinylated Cd200 *Gaussia* luciferase fusion pentamer before coelenterazine was again added to detect interactions. The Cd200-Cd200R interaction was robustly detected over a wide range of bait quantities (1–100 fmole per well in a 96 well plate) (Fig. 3d). Together, these data demonstrate that the pentameric *Gaussia* luciferase reporter enzyme can be used as both a bait and a prey for low affinity extracellular protein interaction screening.

## The *Gaussia* luciferase single protein construct system detects very low affinity interactions in a benchmarked screening assay

**Figure 4:**
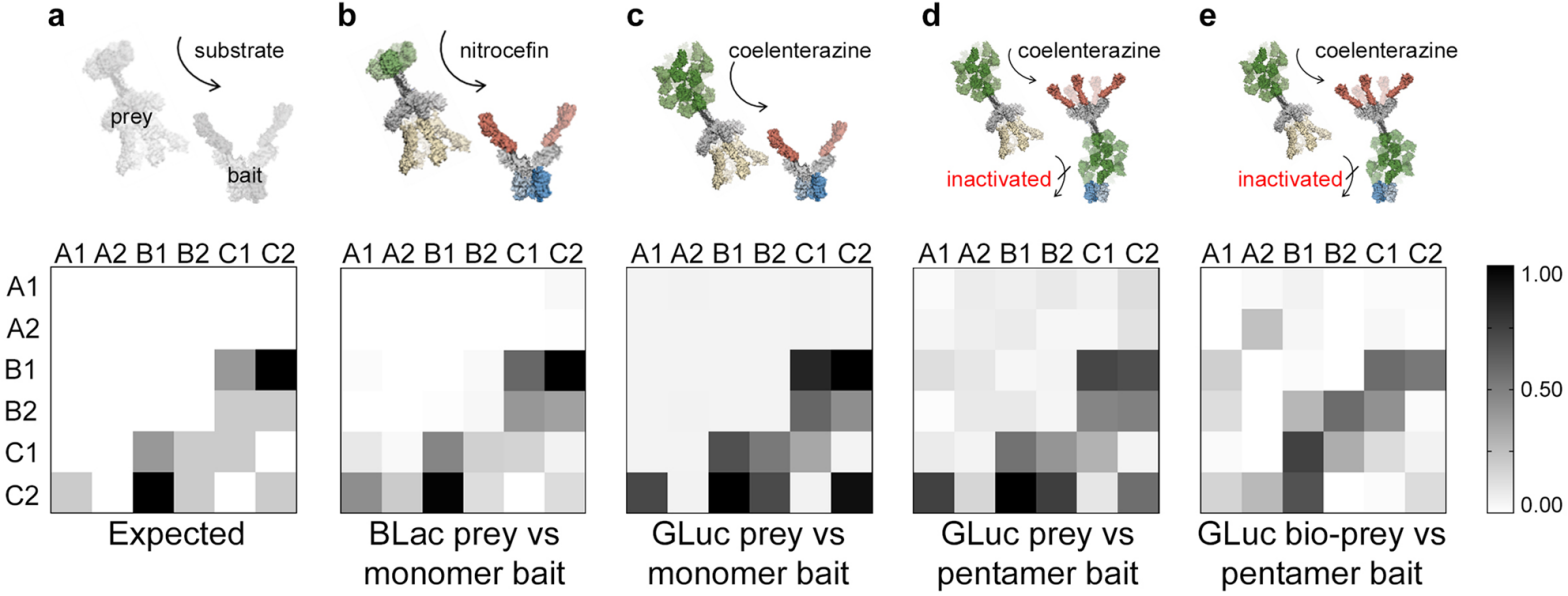
Benchmarking the *Gaussia* luciferase single protein construct system within the zebrafish Jam family protein interactions shows it can detect low affinity interactions with a low false positive rate. The bait and prey versions of the six paralogues of the zebrafish Jam family were expressed and used in different implementations of the receptor interaction assay including both monomer and pentameric *Gaussia* luciferase bait formats. The indicated baits were arrayed on streptavidin-coated plates and systematically probed with the preys for a total of 36 binary interaction tests in each prey-bait combination. (**a**) Graphical representation of expected and observed interactions of different affinities within the zebrafish Jam family. The expected interactions within the zebrafish Jam family are shaded according to their measured dissociation half lives (black = t_1/2_ > 2 s; dark grey 0.4 < t_1/2_ < 2 s; light grey = t_1/2_ < 0.4 s); the scale in (**b**) to (**e**) represents the normalised signal output from the assay. (**b**) Assay read out using the original AVEXIS assay with the beta-lactamase reporter prey against monomeric baits, and (**c**) using the new *Gaussia* luciferase reporter prey against the same array of monomeric baits. In (**d**), the *Gaussia* luciferase pentameric protein is used as a bait, inactivated, and then a same protein construct expressed in a non-biotinylated form as the prey. In (**e**), the same biotinylated *Gaussia* luciferase pentameric protein preparation is used as both a bait and a prey. Representative experiments are shown with *n* = 3 for each prey-bait binary interaction test and summary data are shown in Figure S1.

While the rat Cd200-Cd200R interaction represents a typical extracellular receptor-ligand pair, there are other extracellular receptor interactions that have a significantly lower affinity^4^. To determine the limit of sensitivity of this assay, we used a set of quantified interactions within a family of six paralogous receptors that belong to the zebrafish junctional cell adhesion molecule (Jam) family that have previously been used to benchmark extracellular interaction screening^33^. It is known that the interaction between JamB1 and JamC1 is essential for myoblast fusion in vivo^34^*,* and the interactions within these receptors have been systematically determined and quantified using surface plasmon resonance. These interactions therefore represent a useful set of both homophilic and heterophilic interactions with a range of affinities from the relatively strong JamB1–JamC2 interaction (*t*_1/2_ > 2 s) to the very weak JamA1–JamC2 interaction (*t*_1/2_ < 0.4 s), as well as several known negatives^35^; the expected interactions are depicted graphically in Fig. 4a. Each of the six Jam proteins were expressed as monomeric biotinylated baits, a beta-lactamase pentamer prey, a *Gaussia* luciferase (non-biotinylated) pentamer prey, and a *Gaussia* luciferase pentamer biotinylated bait. The proteins were normalised according to the parameters previously established for optimal interaction detection and all 36 possible interactions tested in a pairwise fashion with each Jam bait arrayed in a streptavidin-coated plate, and all six Jam preys probed against them. The original beta-lactamase prey version of the assay performed as previously described^35^, easily detecting the highest affinity interactions. It also detected all the weaker (*t*_1/2_ < 0.4 s) interactions, although two false positives (JamA1 prey – JamC1 bait, and JamA2-JamC2) were identified at this assay stringency (Fig. 4b). We found that using the *Gaussia* luciferase prey with the monomer bait performed exceptionally well, clearly detecting all interactions, including the very weakest ones with no false positives (Fig. 4c). Using the single plasmid system where the *Gaussia* luciferase pentamer is used as both a biotinylated bait and non-biotinylated prey, the assay performed better than the original beta-lactamase prey detecting all the interactions with only the signal for the weakest JamC1 homophilic interaction overlapping with the distribution of background signals (Fig. 4d). Given the ability to use the *Gaussia* luciferase pentamers as baits, we next asked whether the same biotinylated protein preparations could be used as both a bait and a prey. After the immobilisation of the *Gaussia* luciferase pentamer and inhibition of the enzymatic activity by coelenterazine addition, an additional wash step with a buffer containing free biotin to block all remaining biotin binding sites was performed before again adding the *Gaussia* luciferase pentamer to detect interactions. We observed that the assay performed well for the higher affinity JamB1-JamC2 and JamB1-JamC1 interactions, but did not detect the weaker JamB2– JamC2 interaction in either bait-prey orientation (Fig. 4e). Setting a threshold to detect the weaker interactions led to an increase in false positives suggesting this format of the assay would be best used to detect interactions with interaction half-lives of a second or longer.

## Discussion

Despite their fundamental importance in many biological processes and attractiveness as drug targets, identifying extracellular protein interactions between membrane-embedded receptor proteins still poses technical challenges. A great deal of progress has been made, and especially by using ELISA-style assays that detect direct binary binding events within libraries of receptor ectodomains expressed as soluble recombinant proteins in eukaryotic cells^15–18^. While these assays were designed to systematically screen for interactions between hundreds of different proteins, they are not generally suitable for thousands of proteins, a scale that would be necessary to test the majority of tractable receptor proteins encoded by a mammalian genome. Here, we have made progress on tackling two of the main barriers that have prevented this increase in scale: reducing the amount of protein required per binding test by increasing assay sensitivity, and, importantly, designing the assay so that just one—rather than the usual two—plasmid expression constructs and sample preparations per protein are needed.

The use of the “flash” *Gaussia* luciferase presented the opportunity of not only increasing the sensitivity of the prey reporter, and thereby reducing the amount of protein required, but also, because of the reaction-induced inhibition, using the same protein preparation as both a bait and a prey. Previously, we used monomeric biotinylated bait proteins that were clustered around immobilised streptavidin^18^; however, by taking this approach, we were unable to control the local clustering. By using “pre-clustered” pentameric proteins as the bait, we observed that we could also reduce the amount of immobilised bait protein needed. We established that the inhibition of the luciferase was not easily reversible: re-purifying the protein with extensive washing steps, for example, did not result in any enzyme reactivation. Because the experimental properties of the enzyme inhibition were sufficient for our purposes, we did not investigate the molecular basis of inhibition in any further detail. It is known that Cu^2+^ ions are potent inhibitors of *Gaussia* luciferase^36^ and post-reaction structures of the photoprotein obelin^37^ which also uses coelenterazine as a substrate suggest that the enzyme inhibitor could be a product of coelenterazine catalysis, possibly coelenteramide. Alternatively, coelenterazine oxidation may lead to permanent inactivation of the enzyme due to the formation of inhibitory adducts. Our initial idea was to use HRP as a reporter enzyme that could be inactivated because it is a sensitive enzyme that can simply and irreversibly inhibited by azide^38^, and there are a large number of commercially-available substrates. However, we found that the inclusion of HRP in a pentamer expression construct failed due to poor protein expression that was enzymatically inactive, possibly due to protein misfolding or the lack of an essential co-factor in our HEK293 culture.

Both *Gaussia* and nano-luciferases had several advantages as their use as reporter enzymes in these assays. Both were highly expressed as pentameric oligomers using our mammalian expression system, and were highly sensitive, reducing the protein quantity required for screening. In addition, once expressed, the recombinant protein can be normalised directly in tissue culture medium without the need for further processing. There is scope for optimising this approach further because we did observe some reduction in the signal:noise ratio for the weakest interactions when using the *Gaussia* luciferase pentamers as the bait. This could be due to incomplete luciferase enzyme inhibition or possibly some low level monomer exchange due to the COMP-mediated oligomerisation between the immobilised bait and prey; even with these caveats, this assay format out-performed the original beta-lactamase assay when using a non-biotinylated prey. The performance of the assay was affected when using the same biotinylated protein preparation for both bait and prey suggesting that this assay format would be best deployed by increasing assay stringency and detecting relatively high affinity interactions (*t*_1/2_ > 1.0 s). This increase in background may be due to the exchange of biotinylated prey and baits during the prey incubation step or bait protein stripping with the unconjugated biotin wash used to block all remaining biotin binding sites on the streptavidin-coated plate. Although it doesn’t take advantage of using the one-construct system, we did observe that replacing the beta-lactamase enzyme with *Gaussia* luciferase resulted in very high signal:noise ratios across all the known interactions, including the very weak ones leading to a significant improvement in assay performance. Similarly, while it cannot be inhibited in the same way as the *Gaussia* luciferase, and can therefore only be used as a prey reporter construct, we found that the “glow” properties of the nano luciferase was a significant improvement on the use of beta-lactamase as a prey reporter enzyme. The increased sensitivity coupled with the long-lasting luminescence made screening a large number of assay plates very practical. Indeed, we have recently used this assay format to investigate the receptor-ligand interactions involved in the recognition of hepatocyte host cells by the liver stage (sporozoite) of the malaria-causing parasite *P. falciparum*. Using this implementation of the assay, it was possible to produce sufficient protein for a very large receptor interaction screen (88 parasite ligands versus 182 human receptors) by conveniently transfecting cells in 24-well plates rather than larger (250 mL volume) Erlenmeyer flasks^39^.

Systematically screening for direct interactions using ELISA-style binary binding assays against all ~ 2,000 tractable receptor proteins in a mammalian genome necessitates an assay that has a throughput of ~ 10^7^ interaction tests. Importantly, this assay should be able to detect weak interactions (*K*_D_s ≤ 50 µM) that are a feature of many extracellular interactions^4^. We found that the use of *Gaussia* luciferase as a reporter enzyme in a highly avid pentamer construct both reduced the amount protein required and enabled binary interaction screening using a single protein preparation. These refinements have therefore significantly reduced the amount of resources required and will make large-scale extracellular interaction screening less reliant on access to large-scale protein expression infrastructures.

## Methods

### Recombinant protein expression and purification

All proteins were expressed as secreted proteins by transient transfection of the human HEK293E cell line grown in suspension as described^28^. Enzyme and tag sequences were synthesized by gene synthesis (Geneart) essentially as described^40^. The bait proteins were co-transfected with a plasmid for expression of the *E. coli* enzyme BirA, which monobiotinylates a specific lysine residue in a C-terminal peptide sequence. The transfected cells were incubated for 5 days (in the presence of D-biotin or hemin where specified at 50 μM). The supernatants were harvested by centrifugation at 3000*g* for 20 min and either stored at 4 °C until use or purified. Except for HRP constructs, 2 mM sodium azide was used as a preservative for stored supernatants. Where required, proteins were purified from spent tissue culture media using Ni^2+^-NTA resin using an AKTA pure instrument or HisTrap96 well plates (GE Healthcare) as described previously^41^ or according to manufacturer’s instructions. Briefly, plates were spun to remove storage solutions and washed with binding buffer (20 mM phosphate, 0.5 M NaCl, 20 mM imidazole, pH7.4). Supernatants were loaded, spun, and washed three times with binding buffer before being eluted in 500 µl of elution buffer (20 mM phosphate, 0.5 M NaCl, 200 mM imidazole, pH 7.4).

### Enzyme sequences

The following enzyme sequences were used: *Gaussia* luciferase, accession number: AAG54095 residues K_18_ to D_185_; nano-luciferase, AHH41346 residues V_2_ to T_171_; beta-lactamase WP_125075679 residues H_19_ to W_282_; horseradish peroxidase P00433 residues Q_31_ to S_338_.

### Avidity-based extracellular interaction detection

Binding assays were performed as previously described^28^. Briefly, both bait and prey protein preparations were normalised to activities that have been previously shown to detect transient interactions (monomeric dissociation half-lives of less than 0.1 s) with a low false positive rate^18^. Biotinylated baits that had been purified were immobilised in the wells of a streptavidin-coated 96-well microtitre plate (NUNC). Normalised preys were added, incubated for 1 h at room temperature, washed three times in PBS / 0.1% Tween-20, and once in PBS, after which (for the beta-lactamase constructs) 125 µg/mL of nitrocefin was added and absorbance values measured at 485 nm on a Fluostar Optima (BMG laboratories). This method was modified for the alternative prey constructs to suit their enzyme requirements: for *Gaussia* luciferase, coelenterazine was added at 1 μM (or as indicated); to nano-luciferase, fumerazine was added at 1 μM. Luminescence was measured on a Fluostar Omega (BMG) with no filter and 1 s integration.

### Interaction detection analysis for comparison of different reporter outputs

Each of the enzymes compared in this study has a different output whether colorimetric or luminescent with a different signal intensity. To compare them using a normalised signal output that accounted for the background signal, we used the equation S = (S_obs_−S_min_) / (S_max_−S_min_); where ‘S’ is the ‘fraction of maximum signal’ displayed on data plots, ‘S_obs_’ is the raw signal in a test well, ‘S_min_’ is the average background signal taken from Cd4 negative control bait wells, and ‘S_max_’ is the highest signal recorded for the standard interaction at the highest prey concentration. Using this equation, background signal would be 0, and the maximum signal 1.

### Benchmark assay using the zebrafish JAM family proteins

Each of the six Jam proteins^35^ were expressed as the monomeric biotinylated bait, the beta-lactamase pentamer prey, the *Gaussia* luciferase pentamer prey and the *Gaussia* luciferase pentamer biotinylated bait. Each protein was purified by nickel affinity purification as described^41^ and normalised according to the parameters established in the standard assay for optimal interaction detection: the prey protein of interest at 100 pM; 1 pmol of mono-biotinylated bait; 10 fmoles of biotinylated luciferase pentameric bait. These constructs were then tested for binding in a pairwise fashion with each Jam bait arrayed in a streptavidin-coated plate, and each set of six Jam preys tested for binding, making a total of 36 binary interaction tests for each prey-bait combination. In the case of the pentamer bait an additional inactivation step using an excess of coelenterazine was used followed by a wash before the addition of prey protein. The beta-lactamase prey was assessed using accumulated hydrolysis of the colorimetric substrate nitrocefin, while the *Gaussia* luciferase preys were assessed using luminescence shortly after substrate addition.

## Supplementary information

Supplementary information

## Data Availability

All data generated or analysed during this study are included in this published article, or available from the corresponding author upon reasonable request.
