# Supplementary material for: Rapid and sensitive large-scale screening of low affinity extracellular receptor protein interactions by using reaction induced inhibition of Gaussia luciferase
Source: Sci Rep. 2020 Jun 29;10:10522. doi: 10.1038/s41598-020-67468-7 (PMC7324543; doi:10.1038/s41598-020-67468-7)
Supplement: Supplementary file 1 — Supplementary information [file 41598_2020_67468_MOESM1_ESM.docx]

Supplementary Information for:

Rapid and sensitive large-scale screening of low affinity extracellular receptor protein interactions by using reaction induced inhibition of *Gaussia* luciferase.

Francis Galaway and Gavin J Wright


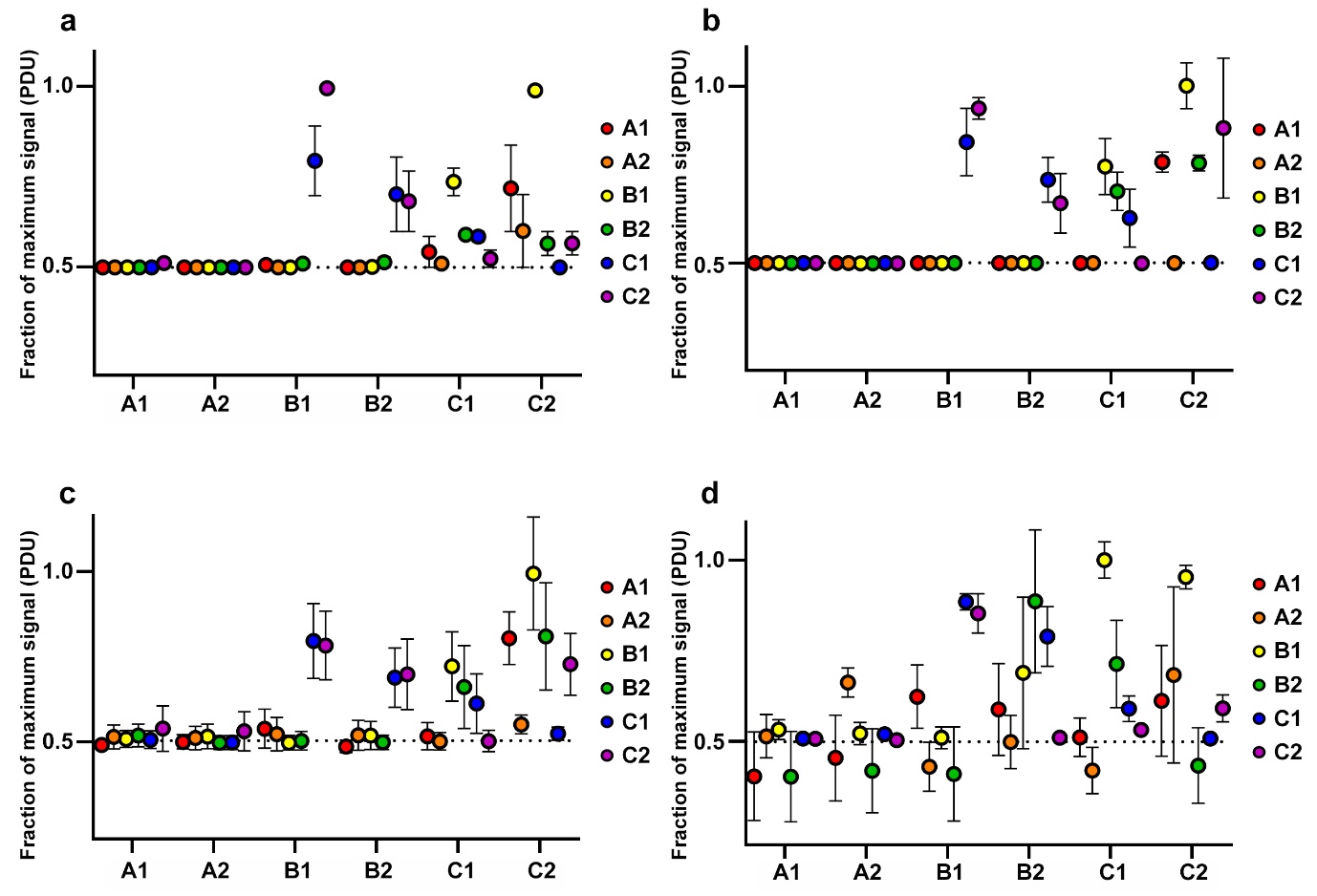


**Figure S1. Summary statistical data for benchmark screening using *Gaussia* luciferase in different screening formats**. The entire ectodomains of the six paralogues of the zebrafish Jam family were expressed as either bait or prey proteins for use in different formats of the receptor interaction assay including both monomer and pentameric *Gaussia* luciferase baits. The different assay formats are: (**a**) beta-lactamase in the original format of the assay; (**b**) monomer bait with *Gaussia* luciferase pentameric prey; (**c**) *Gaussia* luciferase pentamer used as a bait and non-biotinylated pentamer prey; (**d**) and same *Gaussia* luciferase biotinylated pentamer used as both bait and prey. The normalised signal for each data point was calculated as described in the Methods; data points represent means ± s.e.m.; *n* = 3. Each prey is indicated as a coloured circle screened according to the key and screened against the named baits.
